# Editorial: Quality of ornamental crops: effect of genotype, preharvest, and improved production chains on quality attributes of ornamental crops, volume II

**DOI:** 10.3389/fpls.2024.1449585

**Published:** 2024-08-13

**Authors:** Patrícia Duarte de Oliveira Paiva, Margherita Irene Beruto, Renato Paiva, Julian C. Verdonk

**Affiliations:** ^1^ Department of Agriculture, Federal University of Lavras, Lavras, Brazil; ^2^ International Society for Horticultural Science (ISHS), Leuven, Belgium; ^3^ Department of Biology, Federal University of Lavras, Lavras, Brazil; ^4^ Department of Plant Science, Wageningen University and Research, Wageningen, Netherlands

**Keywords:** floriculture, plant physiology, ornamental plant, postharvest quality, stress effects

Considering the interest in the release of a first volume on the Research Topic “Quality of Ornamental Crops: Effect of Genotype, Preharvest, and Improved Production Chains on Quality Attributes of Ornamental Crops”, the Editorial team wanted to collect further findings in a second volume to highlight innovation, technology, and sustainability of ornamental plant production. Indeed, the diversified ornamental sector is characterized by a fast-changing evolution and technological advancements appear to be one of the most important driving forces of the ornamental industry.

This Research Topic collected 10 articles to provide readers with findings aimed to clarify important challenges faced during ornamental plant production. The research and review articles published included information and strategies to set a sustainable production and to get innovative products, including new uses of ornamentals in the urban context. The ornamental sector is considered in its complexity and the published research aims to involve the different levels of production from breeding to postharvest. In the following paragraphs, we summarize the findings of the published articles according to three main topics.

## Production and development aspects of ornamental crops

Urban green spaces are becoming increasingly popular. It is expected that their impact on the population will increase in the coming years because by 2050, 68% of the global population will live in cities. Creating green spaces in urban or peri-urban areas poses challenges related to the condition of the selected space prior to plantation (e.g., physico-chemical characteristics of soil and quality of the environment) and the appropriate land planning and management. Moreover, the choice of appropriate ornamental plants has a pivotal role. The selection of ornamental plants should be based on their aesthetic value and ability to adapt to conditions such as the shading of buildings and trees. A non-destructive leaf gas exchange, chlorophyll a fluorescence, and chlorophyll content are proposed as useful tools for selecting suitable ornamental plants under diverse shading conditions (Francini et al.).

The ornamental sector needs to be fully prepared for high-standard production. The control of environment in greenhouses allows a consistent and high-quality cut-flower production. In particular, photoperiod manipulation is important in managing the production of flowers and improving the productivity of many floriculture crops. Since there was a lack of information on this subject, the research developed by Spall and Lopez indicated a suitable photoperiod process for marigold (*Tagetes erecta*) and witchgrass ‘Frosted Explosion’ (*Panicum capillare*) production, varying the period of the light in different phases of plant development. Moreover, light and gravity can affect plant development by influencing the movements of plant hormones. Transcriptome analysis revealed that stem bending in water lily (*Nymphaea tetragona*) cut flowers is regulated by multiple factors and genes. Differentially expressed genes (DEGs) were associated with the dorsal and ventral stems of the water lily, suggesting a transcription-based regulation of stem bending. Significant differences in genes associated with plant hormones, calcium ions, glucose metabolism, and photosynthesis pathways were observed and related to stem curving, in addition to changes in the cell morphology during the vase life (Li et al.). The authors highlighted that the higher concentration of the auxin indole-3-carbosaldehyde on the dorsal part compared to the ventral part, due to gravity and light, can account for the bending of water lily cut-flower stem (Li et al.).

Regarding abiotic stress factors, drought may be considered one of the most relevant, affecting plant growth and development, and providing serious losses. Transcriptome analysis using an RNA-Seq approach identified unigenes and differentially expressed genes (DEGs) in drought stress in *Heimia myrtifolia*, a native tropical plant. The most prevalent was abscisic acid signal transduction although other plant hormone signal transductions were also involved in the drought stress response. The balance and stability maintenance of the metabolic processes involved many photosynthesis-related and antioxidant enzyme genes (Lin et al.). The findings in this research provide important information for future studies of the drought resistance in other ornamental plants.

## Germplasm characterization and phylogeny

The ornamental sector is characterized by the need for new products. In the next years, it is expected that the demand for floriculture products will increase, and it becomes more evident that consumers value the novelty and sustainability of floriculture products (Gabellini and Scaramuzzi, 2022). In this light, germplasm collection and characterization are of great importance in breeding new cultivars. Some germplasms do not need further breeding and can be used by local growers mainly for the market focused on specialty crops (Darras, 2021). Germplasm characterization refers to the agronomic description of plant materials including traits concerning their aesthetic features and other issues concerning crop improvement (e.g., disease resistance, suitability for a certain production area, or adaptability to climate change). To ensure the best utilization of the germplasm collection for breeding purposes, genetic approaches are also fundamental. In this regard, a study investigated the potential of start codon targeted (SCoT) markers for analyzing genetic diversity among *Cyclamen* species and/or genotypes. Sequence-related amplified polymorphisms (SRAP) markers proved to be useful tools for their separation particularly when combined with phenotypic data. The unweighted pair group method with arithmetic mean (UPGMA) method for hierarchical clustering technique was used to generate the dendrograms. Comparison between the color measurements of flowers and leaves with SCoT analysis revealed differences at species level, discriminating between similar genotypes (Cornea-Cipcigan et al.).

Concerning the transcription factors, the MADS (MCM1, AG, DEFA, and SRF (serum response factor) transcription factors) box transcription factor family and its members play critical roles during plant growth and development. For example, the MIKC^C^-type gene family plays important roles in plant growth, especially during floral organ differentiation. Despite this importance, studies on MIKC^C^-type genes available for roses focused only on transcriptome data, providing an inaccurate mapping and incomplete characterization of these genes. The results of this study provide new insights into the functions of MIKC^C^ genes in rose and provide the basis for future work to explore the evolution of *Rosaceae* (Wang et al.).

Genome editing (GE) methods, particularly those mediated by CRISPR/Cas-related tools are a successful strategy used to alter the function of key genes, regulating biological processes including plant male sterility (MS). These precision breeding technologies can accelerate the line development of new genetic variability with the accumulation of favorable alleles. Farinati et al. provided a general overview of insights and advances in the mechanisms underlying the recent CRISPR technology focusing on plant male sterility applied to main crops and ornamental species.

## Preharvest to postharvest advances in cut flowers

The ornamental industry strongly relies on its final products that are recognized and valued for their aesthetic qualities, thus, it is mandatory for the products to keep their attractiveness also during the postharvest period. After harvesting, different practices have been proposed to prolong the shelf life of ornamentals. This step in the production flow of ornamentals remains challenging. For this reason, it is important to explore the underlying mechanisms related to this delicate phase by promoting a wide perspective on the whole cultivation practices and molecular approaches. Vase-life issues are often linked to only postharvest conditions although preharvest conditions may also affect and reduce the quality of cut flowers. In this sense, Verdonk et al. pointed out the importance of encouraging studies focused on providing information gathered during the cut-flower production process. The role of growth conditions such as irrigation, air humidity, and light quantity and quality, which is essential to increase quality and longevity, is discussed in a review (Verdonk et al.). Studying the complex effects of preharvest conditions on quality illustrates the need for more rapid and reliable phenotyping methods (Verdonk et al.). The use of QTL analysis, candidate gene (CG) mapping, and virus-induced gene silencing (VIGS) proved to be a useful combination to identify possible causal genes and for understanding the molecular mechanisms of the resistance of *Botrytis cinerea* in gerbera’s (*Gerbera hybrida*) preharvest and postharvest conditions (Fu et al.).

Regarding tropical flower postharvest, the ‘low-temperature sensitivity’ restricts transportation together with other traditional cut flowers. Packaging is another issue hampered by shape, size, and weight. The limited advancements in *Heliconia* research highlighted the need for a comprehensive review that consolidates all available information on this species,emphasizing chemical treatments, nano-based technology, and advanced packaging techniques (Malakar et al.).

## Future and outlook

This Research Topic outlined some aspects concerning the ornamentals production. Although many issues still need to be covered in the future, all the publications in this Research Topic present novelty and contribute outstanding information for the ornamental plant development.

In particular, we would like to empathize that important contributions for flower postharvest have been made, and the articles in this Research Topic show that there is a need for research on preharvest, and anatomical and biochemical aspects should be the focus in the future. The results of a meta-analysis on tropical flowers noted that research on identifying solutions to deepen understanding and improve techniques, consequently improving postharvest quality, remains the focus of this industry (Cunha Neto et al., 2023). A future outlook focusing on new phenotyping tools is necessary to quantify the complex interactions between cultivation factors and postharvest performance of cut flowers as indicated by (Verdonk et al.).

Moreover, in this Research Topic, transcriptomics was shown to be a powerful tool in gene identification (Lin et al.; Wang et al.; Li et al.). Future challenges include addressing new potential applications of CRISPR/Cas systems, especially for MS mutant production and transgenerational gene editing for precision-breeding strategies.

We hope that readers find interesting ideas for their research, thus contributing to increasing knowledge on the production of ornamentals, a smaller sector compared to the main agricultural crops but with a significant economic impact.

## References

[B1] Cunha Neto A. R. Paiva P. D. O. Ponce M. M. Calvelli J. V. Barbosa S. (2023). Meta-analysis of new technologies in post-harvest of tropical flowers. Ornam. Hortic. 29, 224–237. doi: 10.1590/2447-536X.v29i2.2643

[B2] Darras A. (2021). Overview of the dynamic role of specialty cut flowers in the international cut flower market. Horticulturae 7, 51. doi: 10.3390/horticulturae7030051

[B3] Gabellini S. Scaramuzzi S. (2022). evolving consumption trends, marketing strategies, and governance settings in ornamental horticulture: a grey literature review. Horticulturae 8, 234. doi: 10.3390/horticulturae8030234

